# Formation of Interstitial Hot-Spots Using the Reduced Gap-Size between Plasmonic Microbeads Pattern for Surface-Enhanced Raman Scattering Analysis

**DOI:** 10.3390/s19051046

**Published:** 2019-03-01

**Authors:** Taeksu Lee, Sanghee Jung, Soongeun Kwon, Woochang Kim, Jinsung Park, Hyungjun Lim, JaeJong Lee

**Affiliations:** 1 Department of Nano Manufacturing Technology, Korea Institute of Machinery and Materials (KIMM), 156 Gajeongbuk-Ro, Yuseong-Gu, Daejeon 34103, Korea; rubin16@kimm.re.kr (T.L.); luckyheeee@naver.com (S.J.); sgkwon@kimm.re.kr (S.K.); hjlim@kimm.re.kr (H.L.); 2 Department of Control and Instrumentation Engineering, Korea University, Sejong 30019, Korea; chang1121@korea.ac.kr (W.K.); shindew@korea.ac.kr (J.P.)

**Keywords:** nano-imprint lithography, SERS, electrodeposition, nano patterning, gold, silver, nano-gap

## Abstract

To achieve an effective surface-enhanced Raman scattering (SERS) sensor with periodically distributed “hot spots” on wafer-scale substrates, we propose a hybrid approach combining physical nano-imprint lithography and a chemical deposition method to form a silver microbead array. Nano-imprint lithography (NIL) can lead to mass-production and high throughput, but is not appropriate for generating strong “hot-spots.” However, when we apply electrochemical deposition to an NIL substrate and the reaction time was increased to 45 s, periodical “hot-spots” between the microbeads were generated on the substrates. It contributed to increasing the enhancement factor (EF) and lowering the detection limit of the substrates to 4.40 × 10^6^ and 1.0 × 10^−11^ M, respectively. In addition, this synthetic method exhibited good substrate-to-substrate reproducibility (RSD < 9.4%). Our research suggests a new opportunity for expanding the SERS application.

## 1. Introduction

Surface-enhanced Raman scattering (SERS) allows for highly sensitive, non-destructive, and molecular-specific detection and has become a powerful tool for biological, pharmaceutical, and environmental detection [1,2,3,4,5]. Since surface plasmon induced electric field enhancement is a key factor in the general enhancement mechanism in SERS, a reliable formation of “hot-spots” is a prerequisite for practical use of SERS to reach the highest value of enhanced electromagnetic field [6]. 

Bottom-up approaches, like self- or guided-assembly of nanoparticles, are able to reduce the distance between metallic nanoparticles down to 1 nm or less and can lead to detection of target molecules at nearly the 1 fM level. Unfortunately, bottom-up methods are usually accompanied by loss of reproducibility, considering the location, density, and signal EF of the “hot-spots”, which prevents the reliable large-scale fabrication of these structures for commercialization [7,8,9,10].

From the viewpoint of practical applications, substrate-based SERS sensors are much more preferable than solution-based sensors due to their robustness and mass-productivity. Hence, many technical top-down lithography approaches, such as electron beam lithography, focused ion beam milling, and nanosphere lithography, have been proposed for efficient SERS substrates with good reproducibility and mass-production. However, it is quite difficult and costly to control the interstitial distance between neighboring plasmonic structures to create plasmonic “hot-spots” [11,12,13].

Here, we report a top-down and bottom-up hybrid method for the formation of “hot-spots” on the wafer-scale. Uniform nanohole patterns were fabricated via nano-imprint lithography (NIL) on the entire substrate [14,15,16], functioning as preferential sites for reducing silver ions under the −2.0 V of over-potential (vs Ag/AgCl) [17] and yielding silver microbead patterns (SMPs). By increasing the electro-deposition time to 45 s, it was possible to generate interstitial “hot-spots” among SMPs. Although other similar investigations have been carried out for SERS sensing [11,12,13,14,15,16], this combined top-down and bottom-up strategy enabled us to achieve good substrate-to-substrate reproducibility (RSD < 9.4%) and a low detection limit (1 × 10^−11^ M) of a well-known Raman probe, Rhodamine 6G (R6G), over the entire chip (3 × 3 cm^2^). Given the advantages of this mass-scalable, fast, simple, and inexpensive process, this fabrication strategy is expected to facilitate practical SERS applications.

## 2. Materials and Methods

### 2.1. Preparation of Nanohole Pattern

To prepare the nanohole pattern, Si (100) wafer was covered with 5 nm Cr and 50 nm Au thin-films using e-beam evaporation. Then, polymethylglutarimide (PMGI) (PMGI SF5s, MicroChem Corp., Westborough, MA USA) was spin-coated on the substrate at 3000 rpm. Then, thermal nanoimprint resist (mr-I 8010R, micro-resist technology GmbH, Berlin, Germany) was spin-coated on the PMGI thin film at 3000 rpm. Using a commercial tool (ANT-6HO3, KIMM, Daejeon, Korea), the bilayer resist stack was subjected to thermal nanoimprinting at 200 °C under a pressure of 30 bar for 200 s with a pillar patterned silicon stamp (150 nm diameter, 1000 nm pitch) as the nanoimprint mold. After the nanoimprinting step, the residual nanoimprint resist was etched by O_2_ plasma, after which the PMGI resist was wet-etched using a commercial developer solution (AZ-MIF300, AZ Electronic Materials, Darmstadt, Germany) for 3 s. 

### 2.2. Synthesis and Characterization of Silver Microbead Patterns (SMPs)

Prior to the electrodeposition process, the nanohole patterns were rinsed with deionized water twice. Non-reactive areas on the substrates were passivated using lacquer for better fixation of the substrates on the working electrode and determining the reactive area for silver nanostructure growth in the nanohole pattern. The lacquer was deposited on the edge of the substrate and dried at room temperature. We conducted electrochemical experiments with a conventional three-electrode system (Pt wire counter electrode and Ag/AgCl (3 M KCl) reference electrode) using a potentiostat (CompactStat, Ivium Eindhoven, The Netherlands). A concentration of 20 mM of silver-plating solution was prepared. Different types of SMPs were synthesized by varying the deposition time (15 to 45 s) under ‒2.0 V of applied overpotential (vs Ag/AgCl). The morphologies of the SMPs, depending on the deposition time, were detected using field emission scanning electron microscopy (FE-SEM, S-4800, Hitachi, Tokyo, Japan) and the averaged diameter of each SMPs were analyzed using the ImageJ program (National Institutes of Health, Bessezda, MD, USA). The crystal structures of the SMPs were investigated using X-ray diffraction (PANalytical B.V., Almelo, Netherland) at room temperature. In addition, the surface elements in the nano-hole patterns (NHP) and SMPs were measured using an X-ray photoelectron spectrometer (K-alpha, Thermo, Waltham, MA, USA) with a monochromatic AlKα X-ray source.

### 2.3. Surface-Enhanced Raman Scattering (SERS) Measurements

For the SERS analysis, we prepared solutions of rhodamine 6G (from 10^−11^ to 10^−4^ M), in ethanol. R6G was purchased from Sigma-Aldrich (Seoul, Republic of Korea) and ethanol was obtained from SAMCHUN Chemical (Seoul, Republic of Korea). The fabricated substrates were immersed in each solution for 1 h and dried at room temperature. Raman spectroscopy measurements were performed with confocal Raman microscopy (Renishaw inVia Raman Microscope, Gloucestershire, UK) under a He-Ne laser (λ = 633 nm). 

The laser power on the sample was approximately 1 mW, and the integration time was 10 s. Before detection, we performed calibration using a silicon wafer with a Raman band at 520 cm^−1^ as the reference. Wire 3.2 software (Gloucestershire, UK) was utilized for spectral and image processing and analysis. 

### 2.4. Calculation of SERS EF

The analytical SERS EF for rhodamine 6G molecules on the SMPs3, selected as the most optimum condition, were calculated using the following equation:

EF = (I_SMPs3_/I_dye_) × (C_dye_/C_SMPs3_) (1)

C_dye_ and C_SMPs3_ represent the treated rhodamine 6G concentration (10^−4^ M) on the bare Si substrate and the treated rhodamine 6G concentration (10^−11^ M) on the SMPs3. Specifically, the bare Si substrate was immersed in a rhodamine 6G solution like SMPs3; I_dye_ and I_SMPs3_ are the signal intensities of the rhodamine 6G Raman spectra on the pure Si substrate (after 10^−4^ M rhodamine 6G solution-treated) and on the surface of SMPs3 (after 10^−11^ M rhodamine 6G solution-treated).

## 3. Results and Discussion

**Scheme 1:**
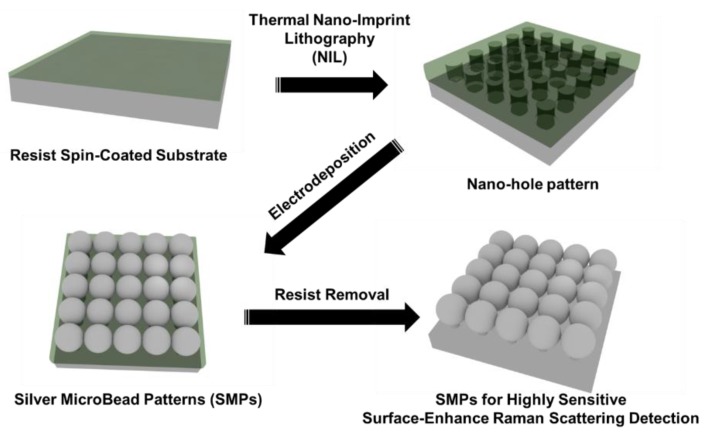
Illustration of overall process in this research. Nanohole arrays were physically defined by nano-imprint lithography. Silver microbeads were generated using electrodeposition for each hole-pattern. Then, interstitial “hot-spots” were generated by increasing the size of silver microbeads for SERS analysis.

**Figure 1:**
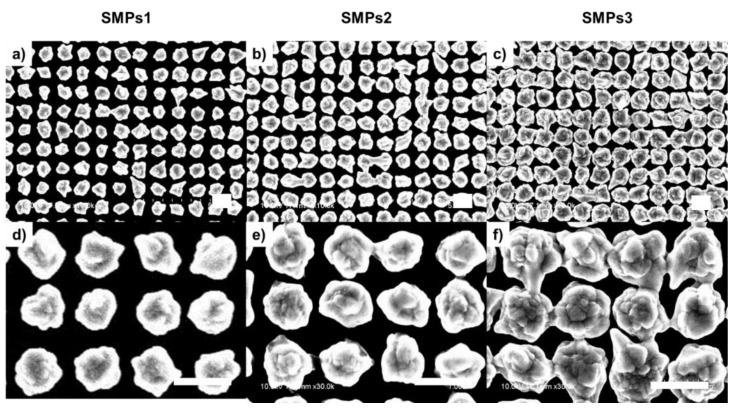
Morphology of SMPs with variation of deposition time. SEM images of SMPs with respect to different electrodeposition conditions. Deposition during 15 s (**a**,**d**), 30 s (**b**,**e**), and 45 s (**c**,**f**). Scale bars are 1 μm.

In order to prepare the interstitial “hot-spots” on the wafer, we used nano-imprint lithography (NIL) and chemical deposition hybrid method, as illustrated in Scheme 1. Uniform nanohole patterns (NHPs) were prepared via NIL. Since the electrochemical deposition process was conducted using the metal surface of substrate, the non-conductive resists should be removed. In order to open preferential deposition sites, the thermal nanoimprint resist was etched using O_2_ plasma [1,16]. During the plasma etching, PMGI under thermal resist shielded the metal substrate from reactive ions. PMGI was selectively removed with AZ-MIF 300 after the NIL process. The top-down procedures of NIL offered benefits of reproducible pattern generation and high-throughput. As part of the bottom-up process, chemical deposition of silver microbead patterns (SMPs) was carried out under −2.0 V of over-potential (vs Ag/AgCl). Figure 1 shows SEM images of a deposited silver structure, depending on the reaction time. At short deposition times (15 s, SMPs1), every nanohole pattern was deposited with a silver structure, but the distance between the SMPs1 was relatively too far to generate “hot-spots” for SERS analysis (Figure 1a,d). When the deposition time increased to 30 s (SMPs2), the distance between the SMPs2 decreased and the formation of “hot-spots” between the silver structures could be confirmed (Figure 1b,e). However, the “hot-spot” density in SMPs2 was notoriously poor because the distance between the silver structures were still large. At 45 s of deposition time (SMPs3), size increment in SMPs3 resulted in the generation of periodical “hot-spots” among the SMPs3, as shown in Figure 1c,f. The averaged diameter of SMPs1, SMPs2, and SMPs3 were about 670 nm, 834 nm, and 1000 nm, respectively. Regardless of the deposition time, SMPs arrays were fabricated because the outsides of the nanoholes were blocked by the non-conductive resist and only the bottoms of the nanoholes were open to the silver precursors. Therefore, SMPs were generated inside nanoholes [1].

**Figure 2:**
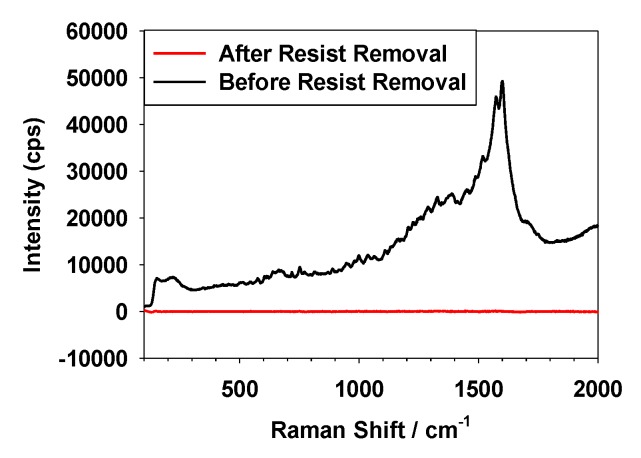
Representative Raman spectra of SMPs3 before and after resist removal.

Prior to the SERS analysis, thermal nano-imprint resist and PMGI in all types of SMPs were removed with an excess amount of acetone and AZ-MIF 300 because they could disguise the target signal (Figure 2).

**Figure 3:**
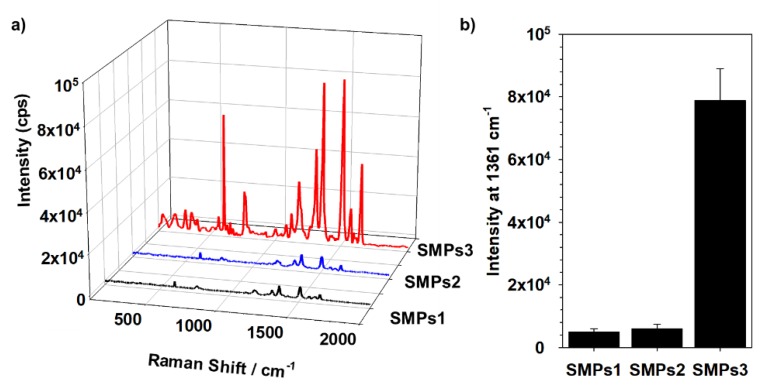
SERS analysis of each SMPs after 10^−5^ M of R6G treatment under 633 nm laser irradiation: (**a**) representative Raman spectra of each SMPs and (**b**) Raman intensity of each SMPs at 1361 cm^−1^. (λ = 633 nm, laser power: 5 mW).

To determine the degree of enhancement of the Raman scattering intensity with respect to the deposition time, we used 10^−5^ M of rhodamine 6G (R6G), a well-known Raman probe, to treat every substrate. In all the SERS spectra (Figure 3a), the most prominent vibrational bands of R6G appeared at 613 cm^−1^(C-C-C in plane bending), 787 cm^−1^ (C-H out-plane bending), 1185 cm^−1^ (C-H and N-H bending), 1312 cm^−1^ (C=C stretching), and 1361 and 1510 cm^−1^ (C-C stretching) [18,19]. Comparing the Raman intensities at 1361 cm^−1^, SMPs1 and SMPs2 revealed small and similar Raman intensities due to the absence or poor density of “hot-spots.” On the contrary, the Raman intensity of SMPs3 at 1361 cm^−1^ was 16.0 and 13.1 times higher than those of SMPs1 and SMPs2 (Figure 3b), respectively, and matched with the existence of “hot-spots”, the reduced nanogap size in SMPs3. The reduced nanogap size was caused by the size increment of silver microbeads, as depicted in the SEM images. In addition, SMPs3 may absorb more molecules because the larger silver structure can absorb the bigger amount of molecules on their surface, Also, the shape of SMPs3 was more spiky than SMPs1 and SMPs2. Thus, SMPs3 present stronger Raman signal intensity than SMPs1 and SMPs2. We chose SMPs3 as the optimal condition for SERS analysis, and further experiments were conducted using SMPs3.

**Figure 4:**
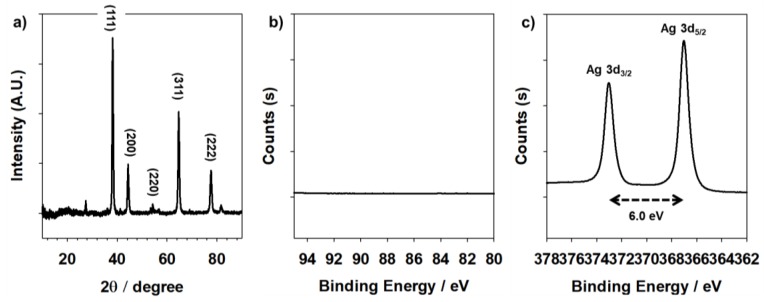
Structural and surface element analysis of SMPs3. (**a**) XRD spectrum of SMPs3. High resolution (**b**) Au 4f and (**c**) Ag 3d XPS spectra of SMPs3.

The X-ray diffraction (XRD) spectrum showed that the synthesized SMPs3 were highly crystalline (Figure 4a). The high-resolution X-ray photoelectron spectra showed the nanohole pattern were fully covered by silver microbeads after electrodeposition because Au 4f peaks were clearly removed in SMPs3 (Figure 4b). Specifically, in the Ag 3d spectrum (Figure 4c), SMPs3 presented doublet peaks of Ag 3d_3/2_ (at 373 eV) and Ag 3d_5/2_ (at 367 eV). The energy difference between the doublet peaks was 6.0 eV and the intensity ratio of these two peaks was 2:3, demonstrating the appearance of silver in its zero-valent form Ag^0^ in the system. Therefore, we could confirm that SERS enhancement of the nanostructures was based on the surface silver composition [1,20].

**Figure 5:**
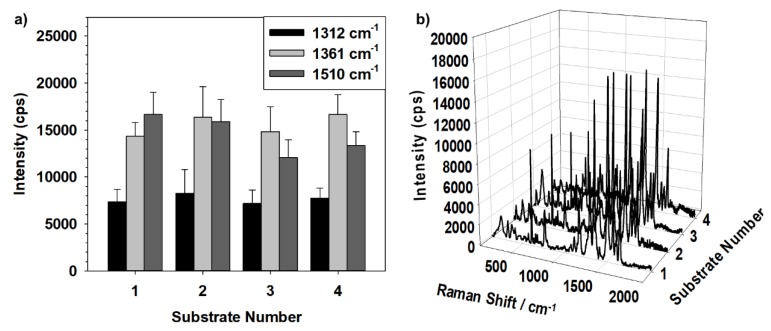
Reproducibility of prepared SMPs3. (**a**) Peak intensities of 10^−5^ M of R6G treated SMPs3 from each synthetic procedure. (**b**) Representative Raman spectra from Figure 5a. (λ = 633 nm, laser power: 5 mW).

Since we used NIL method to fabricate SMPs3, which is well-suited for high throughput and uniformity, we envisaged that the SMPs3 would exhibit a good substrate-to-substrate reproducibility of the SERS signal. Thus, we synthesized four SMPs from different batches in a row and compared the Raman intensity of each sample after treatment of 10^−5^ M of R6G solution. As shown in Figure 5, Raman intensity at distinct Raman peaks of R6G (1312, 1361, and 1510 cm^−1^) from each SMPs3 revealed relatively low standard deviation (RSD < 9.4%) of the average SERS intensities, which demonstrated the synthetic stability of the proposed method.

**Figure 6:**
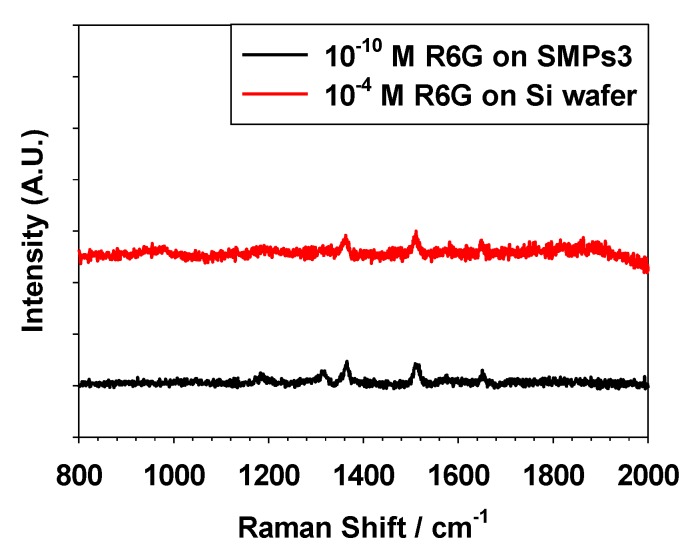
SERS spectra of rhodamine 6G adsorbed on SMPs3 and on planar Si surface. Intensity ratio of two spectra implies an EF of ~10^6^.

To assess the SERS performance of SMPs3, the EF and the variation in SERS intensities with various R6G concentrations were examined. Based on the Raman signal intensity in Figure 6, the analytical EF value of SMPs3 was calculated and found to be 4.40 × 10^6^ using rhodamine 6G (R6G). This EF increment in SMPs was the result of the generation of periodical “hot-spots” on the entire substrate and the silver surface composition on the SMPs (inherently higher SERS intensity than that of gold).

**Figure 7:**
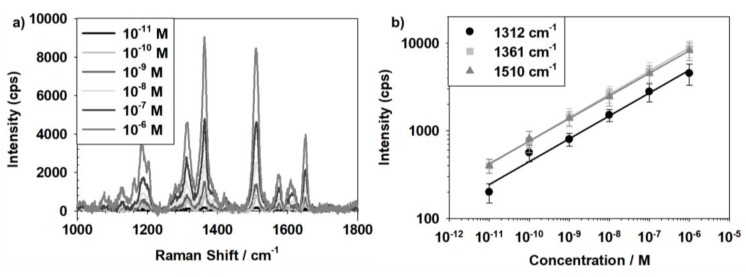
SERS detection limit of SMPs3. (**a**) Raman spectra of SMPs with respect to various concentrations of R6G (10^−11^ M – 10^−6^ M). (**b**) Calibration curves of SERS intensity at 1312, 1361, and 1510 cm^−1^ in SMPs, matched with peaks in R6G. Five random points in SMPs3 were measured at each concentration (λ = 633 nm, laser power: 1 mW).

Figure 7a presents the Raman spectra of SMPs3 after treatment with different concentrations of R6G (1 × 10^−11^ M–1 × 10^−6^ M). The specific peaks of R6G appeared distinctively at each concentration. The SERS intensities of the three different Raman shifts at 1312, 1361, and 1510 cm^−1^ as a function of the R6G concentration were plotted in Figure 7b. The results demonstrate that the Raman intensities of R6G in SMPs3 decreased as the loaded R6G concentration decreased. The slopes at 1312, 1361, and 1510 cm^−1^ were measured and found to be 0.26, 0.27, and 0.26. The limit of detection (LOD) of SMPs3 was defined as the concentration at which the signal to noise ratio was equal to 4. Because we used a 633 nm laser, the LOD of R6G in SMPs3 was identified as 10 pM without the aid of the surface enhanced resonance raman spectroscopy effect.

## 4. Conclusions

Silver microbead patterns (SMPs) for sensitive and reproducible SERS substrates were successfully synthesized by combining nano-imprint lithography and electrodeposition methods. The periodical nanohole patterns were fabricated using nano-imprint lithography and acted as preferential sites for reducing silver ions to generate SMPs. The interstitial distance between the SMPs was narrowed by increasing the electrodeposition time, concomitantly leading to the creation of “hot-spots” that enhanced SERS activity. Since both NIL and electrodeposition method can be scaled up to wafer scale, and the presented results manifest sensitive SERS detection as well as substrate-to-substrate reproducibility, it can be envisioned that our SMPs pattern with interstitial “hot-spots” can be used as a practical SERS substrate in analytical, environmental, and biomedical fields.
